# Molecular Mimicry Between Gut Microbiome and Rheumatoid Arthritis: Current Concepts

**DOI:** 10.3390/medsci12040072

**Published:** 2024-12-12

**Authors:** Anandanarayan Muruganandam, Filippo Migliorini, Naveen Jeyaraman, Raju Vaishya, Sangeetha Balaji, Swaminathan Ramasubramanian, Nicola Maffulli, Madhan Jeyaraman

**Affiliations:** 1 Department of Orthopaedics, Faculty of Medicine—Sri Lalithambigai Medical College and Hospital, Dr MGR Educational and Research Institute, Chennai 600095, India; anandanarayan66@gmail.com; 2 Department of Orthopedics and Trauma Surgery, Academic Hospital of Bolzano (SABES-ASDAA), 39100 Bolzano, Italy; 3 Department of Life Sciences, Health, and Health Professions, Link Campus University, 00165 Rome, Italy; 4 Department of Orthopaedics, ACS Medical College and Hospital, Dr MGR Educational and Research Institute, Chennai 600077, India; naveenjeyaraman@yahoo.com; 5 Department of Orthopaedics and Joint Replacement Surgery, Indraprastha Apollo Hospital, New Delhi 110076, India; raju.vaishya@gmail.com; 6 Department of Orthopaedics, Government Medical College, Omandurar Government Estate, Chennai 600002, India; sangeetha5938@gmail.com (S.B.); swaminathan.ramasubramanian@outlook.com (S.R.); 7 Department of Trauma and Orthopaedic Surgery, Faculty of Medicine and Psychology, University La Sapienza, 00185 Roma, Italy; n.maffulli@qmul.ac.uk; 8 School of Pharmacy and Bioengineering, Keele University Faculty of Medicine, Stoke on Trent ST4 7QB, UK; 9 Centre for Sports and Exercise Medicine, Barts and the London School of Medicine and Dentistry, Mile End Hospital, Queen Mary University of London, London E1 4DG, UK

**Keywords:** molecular mimicry, gut microbiome, rheumatoid arthritis, autoimmune

## Abstract

Rheumatoid arthritis (RA) represents an autoimmune condition impacted by a combination of genetic and environmental factors, with the gut microbiome (GMB) being one of the influential environmental factors. Patients with RA display notable modifications in the composition of their GMB, characterised by decreased diversity and distinct bacterial alterations. The GMB, comprising an extensive array of approximately 35,000 bacterial species residing within the gastrointestinal tract, has garnered considerable attention as a pivotal contributor to both human health and the pathogenesis of diseases. This article provides an in-depth exploration of the intricate involvement of the GMB in the context of RA. The oral–GMB axis highlights the complex role of bacteria in RA pathogenesis by producing antibodies to citrullinated proteins (ACPAs) through molecular mimicry. Dysbiosis affects Tregs, cytokine levels, and RA disease activity, suggesting that regulating cytokines could be a strategy for managing inflammation in RA. The GMB also has significant implications for drug responses and toxicity, giving rise to the field of pharmacomicrobiomics. The composition of the microbiota can impact the efficacy and toxicity of drugs, while the microbiota’s metabolites can influence drug response. Recent research has identified specific bacteria, metabolites, and immune responses associated with RA, offering potential targets for personalised management. However, several challenges, including the variation in microbial composition, establishing causality, accounting for confounding factors, and translating findings into clinical practice, need to be addressed. Microbiome-targeted therapy is still in its early stages and requires further research and standardisation for effective implementation.

## 1. Introduction

Rheumatoid arthritis (RA) is a long-term, systemic autoimmune condition characterised by a gradual onset of symmetric polyarthritis in the hands, wrists, and feet over weeks to months. This condition leads to the gradual deterioration of cartilage and joints and the formation of bone erosions [1,2,3]. A review of international population-based studies calculated the prevalence of RA and looked into how geography, data sources, classification standards, and prevalence definitions affected that figure. On average, the point prevalence of RA is estimated to be 51 cases per 10,000 people, while the period prevalence is around 56 cases per 10,000 people [4]. Nevertheless, there is a notable inadequacy in the availability of comprehensive and current data concerning the impact of RA and its evolving trends in the following years [5]. In cases of long-standing untreated RA, the condition manifests with a combination of systemic symptoms, such as fever, fatigue, and weight loss, as well as a range of extra-articular manifestations affecting various body systems, including haematologic, ophthalmologic, vascular, pulmonary, cardiac, renal, and neurologic systems [6]. The initiation of treatment for RA hinges on several critical factors, including the specific joints affected, the duration of symptoms, and the results of laboratory tests, which typically include rheumatoid factor (RF) and anti-cyclic citrullinated peptide (anti-CCP) antibodies in conjunction with erythrocyte sedimentation rate (ESR) and C-reactive protein (CRP) levels [7,8]. It is imperative to seek aggressive treatment utilising disease-modifying antirheumatic medications, whether they are conventional or biological, in the early stages of RA [9]. About 50% of patients with early-stage RA can achieve remission with methotrexate and a biological disease-modifying antirheumatic medication [10,11]. It is important to note that people with RA have a higher-than-average risk of developing cardiovascular disease, which emphasises the necessity of actively addressing cardiovascular risk factors in this population [12].

The microbiome represents a pool of microbes, either commensal, symbiotic, or pathogenic, with specific properties and specific functions and interactions with the ecological system [13]. Marchesi et al. proposed a microbiome definition based on the genomes, proteomes, and microbial genes expressed in a given environment [14]. The colon hosts the largest population of microbes right from birth. The makeup of this microbial community and its interplay with the host’s immune system are pivotal factors in developing autoimmune and inflammatory diseases [15,16]. Recent studies have provided compelling evidence indicating differences in microbiota composition between individuals with early RA and those without the condition [17]. These findings underscore the significant role of the GMB in triggering polyarthritis and systemic inflammation through various pathways, which may involve mechanisms like molecular mimicry [18,19]. Notably, elevations have been observed in the abundance of *Prevotella copri* in individuals with early RA, highlighting distinct differences in microbiota compositions compared to individuals without RA [20]. This review seeks to explore the complex interplay within the GMB axis and its significance in the context of RA. Additionally, it explores the emerging field of pharmacomicrobiomics and its potential for devising strategies to modulate the microbiome in the context of RA.

## 2. Composition of the GMB

The human GMB, consisting of around 35,000 bacterial species, became the focus of the National Institutes of Health (NIH) in 2007 when they initiated the Human Microbiome Project. This project aimed to investigate and characterise the microbiota found in different human body regions, specifically those associated with four significant species in the human microbiome [21]. A diverse array of bacterial species from different phyla contributes to the healthy composition of the human GMB:

(a) Bacteroidetes: *Sphingobacterium*, *Bacteroides*, *Tannerella*, *Parabacteroides*, *Alistipes*, and *Prevotella*;
(b) Firmicutes: *Lactobacillus*, *Bacillus*, *Clostridium*, *Enterococcus*, *Staphylococcus*, *Ruminicoccus*, *Faecalibacterium*, *Roseburia*, and *Dialister*;
(c) Actinobacteria: This category includes *Corynebacterium*, *Bifidobacterium*, and *Atopobium*;
(d) Proteobacteria: Comprising *Escherichia*, *Shigella*, *Desulfovibrio*, *Bilophila*, and *Helicobacter*;
(e) *Fusobacteria*.

These bacterial species collectively contribute significantly to general well-being and preserving gut health [22,23]. Another critical species in the human GMB is *Verrucomicrobia*, represented by a single member known as *Akkermansia muciniphila*. This bacterium is crucial in preserving a harmonious and beneficial relationship between the microbial community and the host [24,25]. In a healthy GMB, the Archaea phyla (genus *Methanobrevibacter*) has substantial significance in producing methane [26]. The minor contributors to healthy GMB are yeast (*Candida*), macroparasites, viruses, and bacteriophages [22,27,28]. Each species possesses a specific niche for its survival.

## 3. Oral–GMB Axis

**Figure 1:**
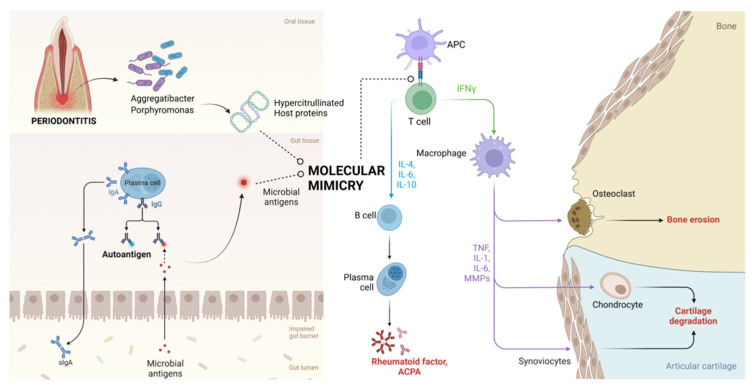
Mechanisms of influence of the oral–GMB axis on the pathophysiology of RA. Oral pathogenic organisms like *Aggregatibacter* sp. and *Porphyromonas* sp. causing periodontitis results in the production of hypercitrullinated proteins that have molecular mimicry with host antigens, thereby contributing to the pathogenesis of RA. Similarly, gut dysbiosis results in the entry of pathogenic microbial antigens that are then presented to the luminal plasma cells, which also possess molecular mimicry to the host antigens, destroying joints, as observed in RA (ACPA: anti-citrullinated protein antibodies; APC: antigen-presenting cell).

RA’s pathophysiology is significantly influenced by the oral–GMB and gut–GMB axes (Figure 1). Through processes including molecular mimicry, bacteria like *Porphyromonas gingivalis* and *Aggregatibacter actinomycetemcomitans* specifically aid in forming antibodies against citrullinated proteins (ACPAs) [29,30]. During inflammation, neutrophils accumulate in affected tissues and may undergo activation or NETosis, releasing PAD (peptidylarginine deiminase) enzymes. These enzymes catalyse post-translational modifications (PTMs), such as citrullination and self-protein carbamylation, creating immunogenic epitopes that bypass immune tolerance and contribute to autoimmunity. Additionally, human PAD and bacterial PAD-like enzymes, such as PPAD from *Porphyromonas gingivalis*, can modify bacterial and host proteins at inflammation sites. These modifications may trigger autoantibody production through molecular mimicry, further promoting autoimmune responses. *P. gengivalis* contributes to the formation of antibodies against citrullinated proteins indirectly through dysbiosis, promoting NETosis, and by directly secreting PPAD [24]. Evidence supports a link between periodontal disease and RA [31,32]. Periodontitis is more likely to occur in people with RA, especially in those who have high levels of anti-CCP antibodies [33,34,35]. Notably, periodontal treatment successfully reduces the symptoms of RA [36,37,38]. The bacterium *P. gingivalis*, the only known bacterial pathogen connected to the pathophysiology of RA, is the source of the enzymes known as PADs [39,40]. Anti-CCP antibody responses in RA patients have been linked to *P. gingivalis* infection [41]. Additionally, *P. gingivalis* has been found in RA patients’ synovial fluid, indicating intracellular location [42].

*P. gingivalis* orally affects the immune system, increasing IL-17 levels in the CIA model [43]. Sato et al.‘s observation of increased Th17 cells in Peyer’s patches and mesenteric lymph nodes [44] lends weight to this conclusion. Notably, when exposed to *P. gingivalis*, IL-17RA-deficient mice showed severe inflammatory bone loss [45]. When exposed to *P. gingivalis*, IL-17RA-deficient animals also experienced severe inflammatory bone loss [46]. Additionally, RA patients have been discovered to have antibodies against *A. actinomycetemcomitans* and its leukotoxin A [47]. In genetically predisposed people, the human PAD4 enzyme and leukotoxin A (LtxA) also promote hyper-citrullination, producing ACPA [48,49]. *A. actinomycetemcomitans* does not encode PAD-like enzymes and seems to drive citrullination by hyperactivating host PADs, which are calcium-dependent, through the activity of LtxA. This toxin promotes prominent calcium efflux, which induces NETosis [50,51]. More studies are required to confirm the part played by *A. actinomycetemcomitans* in the in vivo generation of anti-CCP antibodies [29].

## 4. The GMB and RA

RA is a chronic autoimmune disease marked by systemic inflammation. It often starts with symmetrical polyarthritis that develops gradually and mainly affects the hands, wrists, and feet. It causes bone erosions, joint dysfunction, and cartilage deterioration over many weeks to months [1,2,3]. A complicated combination of genetic and environmental variables leads to the development of RA. Approximately 65% of the risk for RA is a result of genetic predisposition [52]. Genetic factors, particularly the class II MHC genes, particularly HLA-DR, significantly impact RA. The HLA-DR protein’s beta chain has a susceptibility epitope that is crucial in increasing the likelihood and severity of developing RA [53]. RA susceptibility is greatly influenced by human leukocyte antigen (HLA) genes, notably those found in the HLA class II histocompatibility antigen-DRB1-beta chain (HLA-DRB1) gene. This relationship can be shown among several risk alleles for RA, which have a conserved amino acid sequence in common [54,55]. A critical factor in human health and disease is the GMB, a complex collection of bacteria that live in the digestive tract. Its composition may vary according to food choices, antibiotic use, and age [56,57].

According to research, RA and changes in the GMB are closely related. In the setting of RA, studies are actively looking for ways to restore microbial balance [58]. In a retrospective survey of RA patients, researchers found that age and minimal clinically meaningful improvement (MCII) status were the primary factors influencing the variability in GMB composition. Patients who attained MCII demonstrated greater alpha diversity in their GMBs at the beginning and throughout the study period, unlike those who did not achieve MCII. There were notable differences in specific microbial taxa, with *Coprococcus*, *Bilophila* sp. 4_1_30, and *Eubacterium* sp. 3_1_31 being significantly distinctive between the two groups.

Additionally, functional analysis uncovered variations in the abundance of fifteen biochemical pathways, particularly those associated with amino acid and folate biosynthesis. The study also demonstrated the effectiveness of a neural network in predicting MCII status based on GMB profiles, highlighting its potential clinical usefulness [59]. Gender also plays a pivotal role in influencing the function and characteristics of the GMB [60,61,62]. Recent research suggests a link between GMB changes and RA development, as shown in Figure 1 [19,59,63,64,65,66,67].

Numerous crucial mechanisms are involved in the relationship between the GMB and arthritis, including the activation of antigen-presenting cells by Toll-like receptors (TLRs) and NOD-like receptors (NLRs), enzymatic citrullination of peptides, antigenic mimicry, changes in intestinal mucosal permeability, adjustments to the host’s immune system, and the initiation of T helper type 17 (Th17)-mediated mucosal inflammation. These intricate pathways highlight the delicate interactions between the gut flora and the onset of arthritis [68,69]. Autoimmune cells may migrate to the joints following GMB disturbances, harming bone and cartilage [70]. Immune cells are drawn to the synovial membrane, where bacterial antigens produce inflammation. Following the activation of macrophages by autoreactive cells, inflammatory cytokines are released. These cytokines then stimulate fibroblasts, encouraging the synthesis of receptor activators of nuclear factor B ligand (RANKL) and matrix metalloproteinases (MMPs). RA is eventually caused and advanced by these processes, which significantly contribute to the deterioration of bone and cartilage structures [19,67].

## 5. Immunology of the GMB in RA

The connection between the GMB and RA has been closely examined, unveiling the significant influence of changes in the GMB on the immunological processes implicated in RA. Animal models, mainly sterilised (GF) and gnotobiotic mice colonised with specific bacteria, have provided invaluable insights into the role of the GMB in shaping the intestinal immune system. These studies have demonstrated reductions in crucial immune components, such as lymph nodes, Peyer’s patches, Th17 cells, and compromised Treg cells [20,71,72]. The mechanisms by which the GMB influences RA are intricate and pivotal. Dysbiosis in the GMB can initiate a series of immune responses in RA, producing auto-reactive T cells, a critical step in autoimmune reactions. Additionally, the GMB might activate innate immunity, primarily through interactions with fungi and the engagement of critical immune receptors like Toll-like receptors 2 and 4 (TLR2 and TLR4). This interaction might lead to an increase in pro-inflammatory Th17 cells and a decrease in regulatory T cells (Treg cells), disrupting the delicate immune balance required for homeostasis [71,73,74].

Furthermore, the GMB contributes to the production of GPI antibodies and the expansion of Th17 cells within the intestine, leading to the generation of autoantibodies targeting type II collagen and the release of inflammatory cytokines into the bloodstream. Several factors, including specific bacterial species like *Prevotella,* the regulatory influence of butyrate, the role of mucosal immunity, dietary choices, and the use of medications such as sulfasalazine and methotrexate, which have immunomodulatory properties, all contribute to this process [71,74,75]. The metabolic byproducts of the GMB, which consist of fatty acids, have a vital role in influencing the immune response [18,76,77].

As seen before, molecular mimicry is another mechanism implicated in RA pathogenesis involving similarities between bacterial peptides and host antigens or their affinity for host receptors [78,79]. For instance, *E. coli*’s DnaJ protein shares a sequence with HLA-DRB1 epitopes linked to RA and can activate synovial T cells in RA patients. The GMB produces metabolites structurally resembling host molecules, including organic acids, bile acids, and lipids. These substances and bacterial components can mimic host antigens or receptors. Additionally, bacterial cell-to-cell communication, like quorum sensing, can impact host cellular processes. In both RA patients and animal models, evidence shows autoreactive T cells targeting autoantigens that share sequences with specific bacteria, such as *Prevotella*. These T cells can recognise bacterial and human antigens, potentially triggering autoimmune responses. Anaerobic microbes in the human microbiota are considered sources of these molecular mimics, contributing to inflammation in autoimmune diseases like RA [78,80,81,82].

Altogether, these mechanisms emphasise the intricate interactions between the GMB and the immune system in the context of RA, highlighting the necessity for a more profound comprehension of these processes to create targeted therapeutic strategies for this complex autoimmune disease. Furthermore, recent investigations have illuminated the possible use of the GMB as a diagnostic and prognostic instrument for RA. The link between particular bacteria and the clinical characteristics of RA, such as rheumatoid factor, inflammatory markers, and disease activity, has been the focus of investigation. These findings suggest that the GMB may serve as a marker for the onset and progression of the disease [18,67,78,80].

Research has unveiled variations in the intestinal microbiota composition when comparing individuals in the early stages of RA with healthy controls. Notably, these differences include a reduction in specific bacteria from the *Bifidobacterium* and *Bacteroides* genera, alongside a substantial increase in species attributed to the *Prevotella* genus in individuals with early RA [19,83]. Scher et al. discovered that *Prevotella copri*, a specific bacterium found in the GMB, exhibited a strong correlation with new-onset untreated rheumatoid arthritis (NORA) patients. The study findings indicated that an increase in the prevalence of *Prevotella* was linked to a decrease in Bacteroides and a decline in other potentially advantageous microorganisms in subjects with NORA (non-rheumatoid arthritis). Furthermore, the researchers identified specific genes in *Prevotella* that were associated with the disease.

Interestingly, when *P. copri* was introduced to mice, they exhibited an increased susceptibility to chemically induced colitis, implying a possible role for this bacterium in the development of RA [63]. RA patients had reduced gut microbial diversity, which was associated with longer disease duration and higher levels of autoantibodies. Their taxonomic study found a rise in unusual Actinobacteria (phylum) and a fall in numerous taxa in RA patients. Notably, three genera were discovered to be connected to RA: *Collinsella, Eggerthella*, and *Faecalibacterium*. Alpha-aminoadipic acid, asparagine, and the pro-inflammatory cytokine IL-17A were significantly correlated with the abundance of *Collinsella*. *Collinsella’s* significance in modifying intestinal permeability and affecting disease severity was further supported by experimental arthritic research [64]. *Eggerthella lenta* was more abundant in RA patients than controls, metabolising ornithine to produce citrulline and carbamoyl phosphate. While no serum citrulline association was found, RA patients may harbour increased gut citrulline levels, potentially promoting citrullination and antibody production, highlighting its possible role in RA pathogenesis [64]. Certain bacteria, including *Collinsella aerofaciens* (species), *Sedimentibacter*, and *Enterococcus*, are more abundant in RA patients than healthy individuals.

Conversely, *Dorea formicigenerans* (species) is less abundant in RA patients. Furthermore, RA patients exhibit increased activity of an enzyme called arginine deiminase, commonly associated with RA-related genes found in *Collinsella* bacteria. Interestingly, the prevalence of *Collinsella aerofaciens* has been associated with factors such as age, higher levels of anti-citrullinated protein antibodies (ACPA), and smoking in patients with RA. Conversely, there is a decrease in the presence of bacteria like *Sarcina*, 02d06, and *Porphyromonas* in individuals with RA [65]. Liu et al. found that the levels of *Lactobacillus* in the faecal samples of RA patients were significantly higher than those in the control group [66].

Numerous studies have provided insights into the connection between particular bacteria and collagen-induced arthritis (CIA), a widely used model for studying RA. For instance, Brand et al. identified an increase in *Clostridiales* (order) in CIA, indicating their potential involvement in the onset of RA [58]. Indeed, an additional study has provided evidence suggesting the involvement of *Collinsella aerofaciens* in the development and severity of arthritis, particularly in the context of the collagen-induced arthritis (CIA) model. This effect is achieved through a mechanism that involves heightened gut permeability, resulting from the diminished expression of tight junction proteins and the production of metabolites that induce collagen degradation within intestinal epithelial cells (IECs) [59]. Taneja et al. revealed elevated levels of *Parabacteroides* in female subjects with disease resistance compared to their male counterparts within a CIA model of RA [84,85,86].

Our knowledge of the microbiome function in the development of arthritis has considerably increased due to animal studies like the one carried out by Maeda et al. [61]. It has been discovered that genetically vulnerable mice with dysbiosis, characterised by an altered composition of the GMB, develop joint inflammation. *Prevotella* spp. overgrowth in the stomach boosted autoreactive T cells’ susceptibility to arthritis-related autoantigens, resulting in immune-driven joint inflammation. *Prevotella copri* (species) was found in high amounts in some early RA patients [20]. Following gender and age, another study found that RA-associated bacteria may enhance intestinal permeability. Co-culturing or giving RA-associated and non-RA-associated bacteria demonstrated that the latter outcompeted the former. Mice given non-RA-associated bacteria had lower levels of pro-inflammatory cytokines and inflammatory monocytes than untreated mice. Additionally, *E. lenta*, a non-RA-associated bacterium, produced Th17 cytokines in mice that were given treatment, indicating that gut commensals may affect both local and systemic immune responses by affecting gut permeability and immunological regulation [87].

Understanding how the GMB and RA are related sheds light on autoimmune diseases. Zadori et al. examined how non-steroidal anti-inflammatory drugs (NSAIDs) and opioids affect the gut flora, finding that these medications can change its makeup. Mucosal inflammation, modifications in motility, pH, bile acid metabolism, and direct prevention of bacterial growth are examples of potential processes. It is intriguing that the disorders that these medications are used to treat, such as spondyloarthritis (SpA), RA, and neuropathic pain in type 2 diabetes mellitus (T2D), overlap with the microbiome alterations that these medications cause. This highlights the need for more research into the interactions between the microbiome and drugs in treating autoimmune illnesses and pain. Therapy-induced dysbiosis may also restrict therapy effectiveness [88].

Furthermore, dysbiosis in the gut and GMB was discovered in a metagenomic investigation involving RA patients and healthy controls; this dysbiosis partially improved with RA treatment. RA patients could be identified by changes in microbial composition, which could be connected with clinical signs and predict the therapy response. Significant changes in RA patients included a decline in bacteria of the *Haemophilus* genus and an increase in *Lactobacillus salivarius* (species). Along with molecular mimicry of RA-related antigens, the microbiota also exhibited alterations in the redox environment and the metabolism of iron, sulphur, zinc, and arginine. The RA-associated microbiome was altered by disease-modifying anti-rheumatic drugs (DMARD) therapy, but the oral and salivary microbiomes remained inadequate. These results shed light on the impact of treatments on the microbiome in RA and dysbiotic patterns [89].

## 6. Pharmacomicrobiomics and Microbiome-Modulating Strategies in RA

The GMB significantly impacts drug responses, forming the field of pharmacomicrobiomics, which explores the interplay between the GMB, genetics, and drugs. The GMB can metabolise drugs, affecting their absorption and activity. This alters drug effectiveness and safety. Strategies targeting microbial enzymes or microbiota composition can enhance drug efficacy. Metabolites produced by the GMB indirectly influence drug responses via the immune system and metabolism. The GMB is vital in tailoring drug therapies for personalised medicine [90]. Metabolomic studies in humans can uncover novel gut microbial metabolic processes. The human GMB is critical in transforming xenobiotics, which enter the body through various routes. Gut microbes can metabolise various functional groups found in xenobiotics [91,92,93,94]. The emerging field of pharmacomicrobiomics suggests that gut microorganisms and their byproducts can affect the absorption, efficacy, and side effects of drugs for inflammatory arthritis. This offers promise for integrating microbiome data into precision medicine in rheumatology, potentially improving personalised treatment [90,95,96,97]. Low-dose methotrexate (MTX) is a standard treatment for RA, but its effectiveness varies among patients, and some experience adverse effects. Predicting patient response is challenging [98,99]. The GMB is crucial in this variability, as MTX dosage affects its composition. Pharmacomicrobiology explores these interactions to understand how the GMB influences drug responses like MTX. This field promises personalised treatment insights by considering the intricate interplay between drugs and the microbiome for RA and other conditions [100]. Artacho et al. studied the human GMB’s ability to predict MTX effectiveness in new-onset RA patients. They identified bacteria and genes linked to clinical responses, especially those involved in purine and MTX metabolism. Using machine learning, they created a microbiome-based model that accurately predicted non-response to MTX treatment in a separate group of patients [101]. A recent study delved into the relationship between the GMB, treatment response, and intestinal dysbiosis in RA. Conventional synthetic disease-modifying anti-rheumatic drugs (csDMARDs) influenced specific bacterial species, including *Lactobacillus* and *Bacteroides fragilis*, in a dosage-dependent manner. The study also linked the *Lactobacillus* to *Porphyromonas gingivalis* ratio to disease activity and RA’s interleukin-17A (IL-17A) levels. Interestingly, those with secondary non-response to csDMARDs had higher serum levels of intestinal fatty acid-binding protein 2 (IFABP2), suggesting a potential link between intestinal permeability and RA treatment response. These findings indicate that GMB balance, bacterial ratios, and intestinal permeability can impact the RA treatment response, offering potential insights for personalised therapeutic approaches [102].

Probiotics promise to influence intestinal microbiota and the immune response, potentially regulating the immune system and disease severity. Etanercept (ETN), which suppresses the immune system, affects the intestinal microbiota composition in treated patients. A systematic review and meta-analysis of 34 trials explored probiotic supplementation’s safety and efficacy in treating inflammatory arthritis. Results showed that probiotics reduced C-reactive protein (CRP) levels in RA patients, suggesting a complementary approach to managing inflammation in this condition [74,103]. The elastic characteristics of the GMB make it an attractive target for enhancing drug effectiveness and safety. This field is critical for the development of personalised medicine as well as the improvement of drug efficacy and safety [90].

## 7. Current Research on the GMB in RA

The complex connection between the GMB and RA has recently attracted much attention. Much research is being done to understand better how changes in the gut microbial ecosystem can affect how RA develops, how severe it becomes, and how well it responds to treatment. This burgeoning field has enormous promise for improving our comprehension of the underlying mechanisms behind RA and directing more specific and efficient therapy approaches for autoimmune illnesses. Long et al. undertook a thorough assessment of recent developments in understanding immune dysregulation across diverse diseases, emphasising the function of microbiome alterations and gut barrier integrity in the systemic inflammation of rheumatic disease. In-depth discussions of cytokine profiles, T cell activation, and possible treatments that target T follicular regulatory cells are included in their study.

Along with alternate B cell depletion methods, the study also examines the significance of age-associated B cells (ABCs) in diseases such as common variable immunodeficiency disorder (CVID), RA, systemic lupus erythematosus (SLE), and HIV. It also emphasises the role of bacterial DNA in immunological activation, soluble CD14 as a measure of monocyte activation, and the role of mutant Bruton’s tyrosine kinase (BTK) in X-linked agammaglobulinemia (XLA) patients’ reduced consequences [104].

Zhao et al. highlighted the significant roles of dysbiosis, metabolic pathways, lipid metabolism, and oxidative phosphorylation in immune regulation and their involvement in autoimmune diseases, such as systemic lupus erythematosus (SLE), rheumatoid arthritis, and Sjögren’s syndrome. In the context of SLE, their findings revealed reduced gut microbiota diversity and a lower *Firmicutes*/*Bacteroidetes* (F/B) ratio in patients, indicative of a disrupted microbial balance. They identified a set of six microbial taxa with differential abundance between active and inactive disease states. *Lactobacillus reuteri* (species) was shown to promote type I interferon production, contributing to SLE pathogenesis, while other *Lactobacillus* species were implicated in the onset and progression of the disease [105]. Rasouli-Saravani et al., in contrast, concentrated on short-chain fatty acids (SCFAs) generated by the GMB, including acetate, butyrate, and propionate. Numerous autoimmune diseases, including type 1 diabetes mellitus (T1DM), multiple sclerosis, inflammatory bowel disease, RA, celiac disease, and systemic lupus erythematosus, are associated with changes in these metabolites. These results highlight the complex relationship between the GMB and autoimmune disorders, predominantly mediated by metabolites [106].

The gut–joint axis in RA was thoroughly investigated by Dagar et al., who also looked into the possibility of altering the GMB as a potential treatment for RA. Their review outlined the state of knowledge on the connection between the GMB and RA and suggested different ways to affect the GMB as a prospective therapeutic approach. They highlighted the importance of including GMB modification as a valuable therapeutic option for managing RA [107].

Nii et al. examined the complete genome sequences of *P. copri* strains and identified a unique genomic region in strains associated with rheumatoid arthritis (RA). In arthritis models, *P. copri* from RA patients induced more severe arthritis than strains from healthy controls, suggesting that certain *P. copri* strains may contribute to RA aetiology by modulating immune responses [108]. A study by Amend et al. investigated serum biomarkers and antibody responses to *Prevotella* species in people with various stages of rheumatic illnesses. Although there was a small link between total blood IgG levels and IgG antibody responses to *P. copri*, no connection was found between these antibody responses and the presence of *P. copri* in the intestine. These results imply that the onset or course of rheumatic disease may not directly correlate with antibody responses to *Prevotella* species [109].

Koh et al. analysed the gut microbiome of 94 RA patients and 30 controls, revealing distinct microbiota in RA. Young RA patients (<45 years) had reduced diversity. Microbiome composition was unaffected by disease activity or most DMARDs. A combination of *Subdoligranulum* and *Fusicatenibacter* predicted good responses to second-line csDMARDs [58]. Liu et al. investigated the effects of *Acanthopanax senticosus* polysaccharide (ASPS) in RA patients. They discovered that ASPS altered the GMB and caused anti-rheumatic effects transmitted through faecal microbiota. ASPS acted by enhancing the expression of γ-glutamylcysteine (GGC) synthetase and promoting its higher expression in blood. Moreover, the metabolite GGC reduced NLRP3 inflammasome activation. Antibiotics prevented this effect, highlighting the pivotal significance of gut bacteria in ASPS-mediated advantages [110]. In their study of the gut–joint relationship in colitis-related inflammatory arthritis, Shon et al. discovered that certain gut bacteria, such as *Bacteroides vulgatus*, exhibited anti-arthritic effects and that propionate synthesis played a part [111]. Additionally, studies have shown that inflammatory arthritis alters the GMB’s circadian rhythms, affecting gene expression and gut barrier integrity. Circadian disturbance impacts gut function and is controlled by intestinal epithelial cell clocks involving REV-ERBs, ROR proteins, and cyclic IgA production [112]. These results highlight the critical role that the GMB could have in RA prediction and treatment planning. More studies are required to thoroughly understand the intricate relationships between the GMB and RA, to clarify the underlying mechanisms, and to examine the potential of microbiota-based therapies in clinical practice.

## 8. Navigating Challenges in GMB Research

While the GMB has become a promising field of research for gaining insights into the development of RA, it is crucial to recognise the limitations associated with studying the GMB in the context of this multifaceted autoimmune disease. One challenge is the variation and need for more consistency in the GMB composition among those affected by RA. Microbial profiles vary significantly between individuals, making it difficult to identify consistent microbial signatures or biomarkers for RA. *P. copri* has also been identified in a minority of healthy individuals in several cohorts, including the Human Microbiome Project and the European MetaHIT project, and found in RA patients [63,113,114,115]. Moreover, establishing a definitive cause-and-effect relationship between alterations in the GMB and RA is challenging. It remains unclear whether GMB dysbiosis precedes or follows the onset of RA. To achieve more profound comprehension of the intricate link between the gut microbiome and the pathogenesis of RA, it is imperative to conduct longitudinal investigations and intervention trials. Such studies are pivotal for unravelling the temporal dynamics inherent in this relationship and determining whether interventions to alter the GMB can influence the onset and advancement of RA [116]. Another obstacle to consider is the existence of confounding variables and variability. Factors such as diet, medications (including DMARDs), the level of disease activity, and the presence of other health conditions can all influence the makeup of the GMB in individuals with RA. Controlling for these variables and accounting for heterogeneity within the RA patient population present significant challenges in interpreting and comparing study results [58,112,117,118,119]. Ultimately, there are challenges when applying GMB research in clinical settings. These challenges include the absence of standardised protocols, variations in sequencing methods, and limited comprehension of the functional consequences of specific microbial alterations [120] We need further validation and large-scale clinical trials to evaluate the effectiveness and practicality of targeting the GMB for therapeutic interventions in RA [121,122].

## 9. Conclusions

Microbiome-targeted therapy (MTT) is still in its infancy, facing several challenges that need to be addressed, including defining a healthy GMB, sequencing and analysing the standard GMB, ensuring the safety and efficacy of GMB interventions, understanding the pharmacomicrobiomics involving the kinetics and dynamics of the GMB, and accounting for individual-specific dynamics in MTT. As researchers delve into these complex issues, they aim to pave the way for the more robust and effective use of MTT, potentially revolutionising healthcare and treatment strategies. The potential of MTT to transform the landscape of RA treatment is promising, offering a beacon of hope for patients and healthcare professionals alike. Given the diverse composition of the GMB and the resulting variation, researchers and clinicians have encountered inconsistent findings in cases of RA. It is advisable to conduct large-scale randomised controlled trials to establish a comprehensive strategy for understanding the role of the GMB in RA and other inflammatory diseases. The flexibility of the GMB enables the deliberate alteration of intestinal microbiota linked to autoimmune and inflammatory conditions, leading to the exciting possibility of developing new therapeutic and interventional approaches for patients with RA.

## Data Availability

No data was generated for this manuscript.
